# Characteristics and Outcome of Post-Transplant Lymphoproliferative Disorders After Solid Organ Transplantation: A Single Center Experience of 196 Patients Over 30 Years

**DOI:** 10.3389/ti.2022.10707

**Published:** 2022-12-14

**Authors:** Vibeke K. J. Vergote, Christophe M. Deroose, Steffen Fieuws, Wim Laleman, Ben Sprangers, Anne Uyttebroeck, Johan Van Cleemput, Gregor Verhoef, Robin Vos, Thomas Tousseyn, Daan Dierickx

**Affiliations:** ^1^ Department of Hematology, University Hospitals Leuven, Leuven, Belgium; ^2^ Department of Nuclear Medicine, University Hospitals Leuven, Leuven, Belgium; ^3^ Interuniversity Institute for Biostatistics and Statistical Bioinformatics, KU Leuven—University of Leuven, Leuven, Belgium; ^4^ Department of Liver and Biliopancreatic Disorders, University Hospitals Leuven, Leuven, Belgium; ^5^ Laboratory of Molecular Immunology, Department of Microbiology, Immunology and Transplantation, Rega Institute, KU Leuven, Leuven, Belgium; ^6^ Department of Nephrology, University Hospitals Leuven, Leuven, Belgium; ^7^ Department of Pediatric Hemato-Oncology, Department of Oncology, University Hospitals Leuven, KU Leuven, Leuven, Belgium; ^8^ Department of Cardiology, University Hospitals Leuven, Leuven, Belgium; ^9^ Department of Respiratory Medicine, University Hospitals Leuven, Leuven, Belgium; ^10^ BREATHE, KU Leuven, Leuven, Belgium; ^11^ Department of Pathology, University Hospitals Leuven, Leuven, Belgium

**Keywords:** epidemiology, transplantation, outcome, prognosis, post-transplant lymphoproliferative disorder, Epstein Barr Virus

## Abstract

Post-transplant lymphoproliferative disorder (PTLD) is a rare but life-threatening complication after transplantation. In this retrospective, monocentric study we aimed to collect real life data regarding PTLD and determine the role of Epstein Barr Virus (EBV) status and year of diagnosis on prognosis. We identified 196 biopsy-proven PTLD after solid organ transplantation (SOT) diagnosed at the University Hospitals Leuven (Belgium) from 1989 to 2019. EBV status was positive in 61% of PTLD. The median overall survival (OS) was 5.7 years (95% CI: 2.99–11.1). Although EBV positivity was not significantly correlated with OS in multivariate analyses (HR: 1.44 (95% CI: 0.93–2.24); *p* = 0.10), subgroup analysis showed a significantly better median OS for EBV negative post-transplant diffuse large B-cell lymphoma (DLBCL) compared to EBV positive post-transplant DLBCL (8.8 *versus* 2.5 years respectively; *p* = 0.0365). There was a significant relation between year of PTLD diagnosis and OS: the more recent the PTLD diagnosis, the lower the risk for death (adjusted HR: 0.962 (95% CI: 0.931–0.933); *p* = 0.017). In conclusion, the prognosis of PTLD after SOT has improved in the past decades. Our analysis shows a significant relation between EBV status and OS in post-transplant DLBCL.

## Graphical Abstract

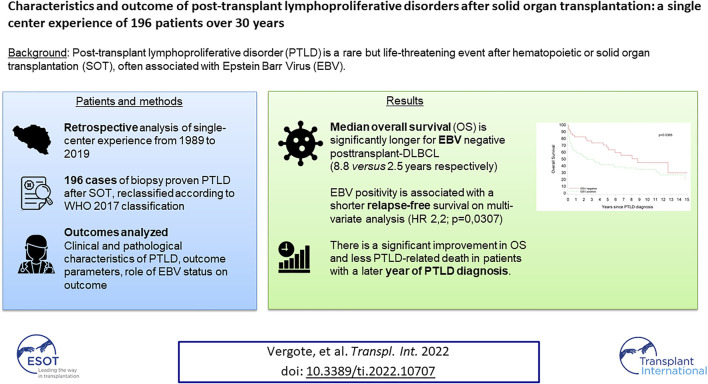

## Introduction

Post-transplant lymphoproliferative disorders (PTLD) are a heterogeneous group of lymphoid neoplasms following solid organ transplantation (SOT) and hematopoietic stem cell transplantation (HSCT)(1,2). The cumulative incidence of PTLD is estimated at 1% after 5 years and 2.1% after 10 years in adult kidney (-pancreas) transplant recipients (3). The risk of developing PTLD depends on the type of organ transplanted and incidence density (i.e. incidence adjusted for time under immunosuppression) ranges from 1.58 per 1000 person-years (kidney), up to 2.24 (heart), 2.44 (liver) and 5.72 (lung) (4-6). The pathogenesis is complex, but two major contributing factors are recognized. Firstly, most cases (60-70%) are associated with infection with the oncogenic Epstein-Barr Virus (EBV) (7-9). Secondly, there is a diminished T-cell immune surveillance due to the iatrogenic immunosuppression in transplant recipients (4,5). The pathogenesis of EBV negative (EBV(-)) PTLD remains the subject of debate. Several hypotheses have been suggested such as the “hit-and-run” hypothesis (where EBV initiates lymphomagenesis, but is then cleared), the role of other viruses (Cytomegalovirus, Human Herpes Virus 8...), chronic antigenic stimulation and long-term immunosuppression(4,10).

The World Health Organization (WHO) 2017 classification recognizes four types of PTLD (1): Non-destructive lesions (2); Polymorphic PTLD (3); Monomorphic PTLD (including B-, T- and natural killer (NK)-cell types) (4); classic Hodgkin lymphoma PTLD (2). Historically, PTLD represents a serious and potentially life-threatening complication of transplantation, with a reported survival rate of 60% after 5 years in kidney transplant recipients (3,5).

Several single- and multicenter reports have previously been published (11-14). However, they are often hampered by their heterogenous population and limited numbers of patients. Large reports from national registries often contain many more cases, but lack detailed information (3,15). Furthermore, significant progress has been made in the past 30 years including a new WHO 2017 classification and improvement of treatment by the introduction of monoclonal antibodies against CD20. Although genomic and transcriptional studies have recently demonstrated that EBV positive (EBV(+)) and EBV(−) PTLD carry different genomic signatures, the role of EBV status on prognosis remains unclear and patients are essentially treated the same (16,17).

Here, we describe one of the largest retrospective single-center series of PTLD after SOT, comprising 196 patients with histologically proven PTLD over a 30 year period. We previously reported our experience in PTLD, including 122 cases after SOT and 18 after HSCT (18). The goal of this report was to analyze a larger group of PTLD after SOT with longer follow-up. We aimed to investigate the role of EBV status on prognosis on a large real life cohort of PTLD and to find out whether prognosis has improved in the past decades.

## Materials and Methods

### Data Collection

**FIGURE 1:**
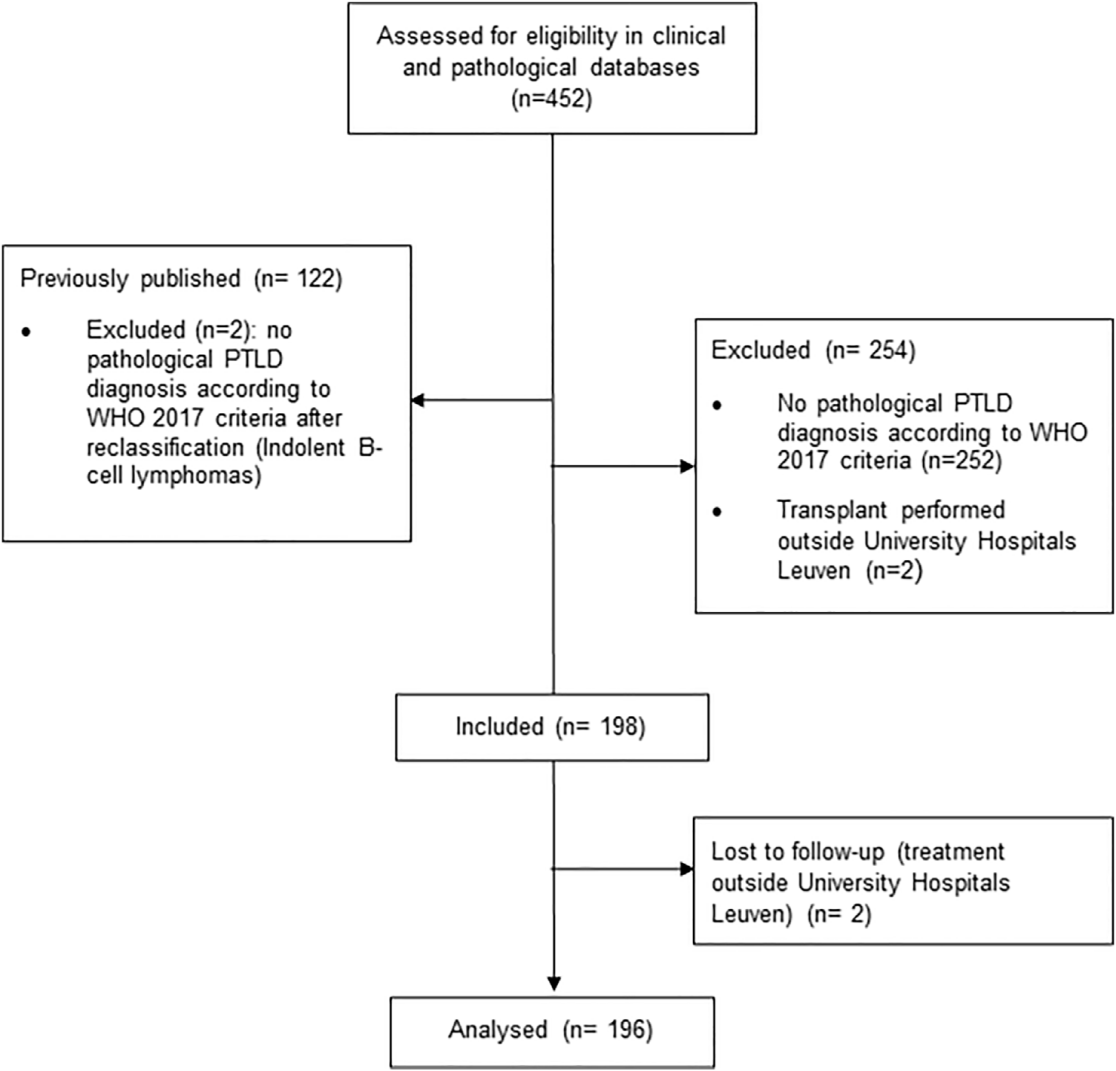
CONSORT flow diagram.

This study was performed at the University Hospitals Leuven (Belgium), a tertiary hospital where all categories of SOT are performed. We reviewed all cases of histologically confirmed untreated PTLD after SOT, diagnosed in our hospital between January 1st, 1989 to December 31st, 2019 (Figure 1). Cases of indolent non-Hodgkin lymphoma (NHL) histology (*n* = 2), with the exception of EBV(+) marginal zone lymphoma, were excluded from analysis, since they are not included in the current WHO 2017 PTLD classification (2). All cases were reviewed by one expert hematopathologist (TT). Patient-related clinical characteristics included gender, age at PTLD diagnosis, Eastern Cooperative Oncology Group Performance status (ECOG PS) and pretransplant EBV serology. Transplant-related characteristics included type of organ transplanted, time from transplantation to PTLD diagnosis and type of immunosuppression. PTLD-related characteristics included: Ann Arbor Stage (19) at diagnosis, presence of B-symptoms, biochemical data (hemoglobin, creatinine clearance, albumin, lactate dehydrogenase (LDH)), number of extranodal sites involved, graft involvement and involvement of different organ systems, (sub)type of PTLD according to the WHO 2017 classification (2), presence of CD20 expression and EBV in the biopsy, year of PTLD diagnosis and data on treatment and outcome variables. If available, data on EBV polymerase chain reaction (PCR) in peripheral blood were collected. This study was approved by the Ethics Committee of University Hospitals/Catholic University Leuven (Ref: S62704 and S55498) and was conducted according to the ethical principles of the World Medical Association Declaration of Helsinki (20).

### Definitions

All PTLD cases required histopathological confirmation to be included. EBV in the biopsy was determined by Epstein-Barr-encoded RNA (EBER) *in situ* hybridization (ISH). Post-transplantation EBV surveillance was not performed systematically in our hospital. International Prognostic Index (IPI) was calculated as previously described (21).

For statistical reasons, patients with combined SOT were pooled according to the transplantation requiring the highest degree of immunosuppression. Patients with combined kidney-pancreas (*n* = 6) and kidney-liver (*n* = 3) were classified as kidney transplantation. Patients with combined heart-lung (*n* = 3) and liver-lung (*n* = 1) transplant were classified as lung transplantation. Lastly patients with combined heart-kidney (*n* = 1) and combined liver-pancreas (*n* = 1) were classified as heart and liver transplantation, respectively.

Response assessment after treatment was performed according to the Lugano criteria (22) and was based upon chart review of the available imaging protocols of computed tomography (CT) or positron emission tomography with ^18^F-fluorodeoxyglucose combined with CT ([^18^F]FDG-PET/CT), if possible including Deauville criteria (23). Timing of response assessment depended on the predefined initial treatment, e.g., after four cycles of rituximab for risk-stratified sequential treatment (24) and after four cycles of rituximab and four cycles of R-CHOP (rituximab, cyclophosphamide, doxorubicine, vincristine and prednisolone) for sequential treatment(25). OS was calculated as time from biopsy-proven diagnosis till the date of death. Death was considered to be PTLD-related in any case where death was caused by either disease progression or a treatment-related complication. Relapse-free survival (RFS) was defined as time from biopsy-proven diagnosis till the date of relapse or death.

### Statistical Methods

A description of the statistical methodology can be found in the Supplementary Material.

## Results

### Epidemiology

**FIGURE 2:**
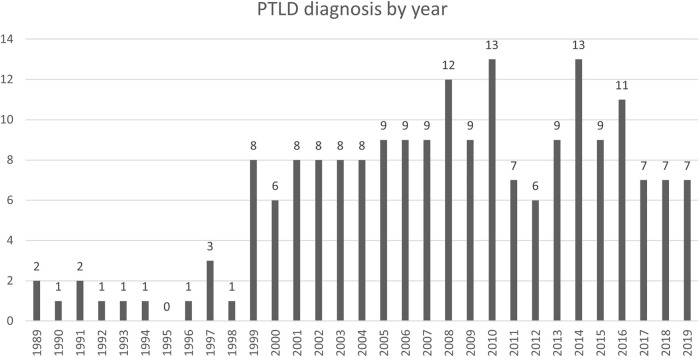
Absolute number of PTLD diagnosis by year.

Between January 1st, 1989 and December 31st, 2019, 7497 patients received a SOT at our center. We identified 196 histologically confirmed cases of PTLD after SOT in the same period. Seventeen patients were pediatric (<18 years) and 179 were adults at time of PTLD diagnosis. There was a male predominance in the adult transplant recipients (58.3%), as well in the PTLD cohort (65.3%). We observed 19 (first decade: 1990–1999), 86 (second decade: 2000–2009) and 89 cases (third decade: 2010–2019), showing a significant increase from the first to the second decade (*p* < 0.0001) and stable number from the second to the third decade (*p* = 0.97) (Figure 2).

### Patient-, Transplant- and PTLD- Related Characteristics

Baseline patient characteristics are summarized in Table 1. The most common transplanted organs were kidney (*n* = 76; 38.8%), lung (*n* = 46; 23.5%), heart (*n* = 30; 15.3%) and liver (*n* = 29; 14.8%). EBV serology before transplantation was negative in 39/96 (40.6%) and positive in 57/96 patients (59.4%) with available data.

**TABLE 1:** Baseline patient characteristics of 196 patients with biopsy-proven PTLD after SOT.

		Years or number (%)
Age at diagnosis (years)	Median (IQR)	54.1 (35.2-64.5)
	Range	3.5-83
Age at diagnosis	≤60 years	122 (62.2)
	>60 years	74 (37.8)
Gender	Male	128 (65.3)
	Female	68 (34.7)
ECOG PS	0-1	138 (70.8)
	2	42 (21.5)
	3-4	15 (7.7)
	Unknown	1
Transplanted organ	Heart	30 (15.3)
	Kidney	76 (38.8)
	Lung	46 (23.5)
	Liver	29 (14.8)
	Heart-Kidney	1 (0.5)
	Kidney-Pancreas	6 (3.1)
	Kidney-Liver	3 (1.5)
	Heart-Lung	3 (1.5)
	Liver-Lung	1 (0.5)
	Liver-Pancreas	1 (0.5)
IS at diagnosis	CNI	189 (96.4)
	AM	152 (77.6)
	CS	134 (68.4)
	Sirolimus	1 (0.5)
	CNI + AM + CS	99 (55.5)
	Induction	94 (48%)
Time between transplantation and PTLD (years)	Median (IQR)	4.3 (1.0-10.6)
	Range	0.2-28
Pathology	Non-destructive	16 (8.2)
	Polymorphic	11 (5.6)
	Monomorphic	162 (82.7)
	Hodgkin	6 (3.1)
	EBV(+) mucocutaneous ulcer	1 (0.5)
EBV ISH at diagnosis	Negative	67 (26)
	Positive	119 (64)
	Unknown	10
CD 20 expression at diagnosis	Negative	31 (16.1)
	Positive	155 (80.3)
	Partially positive	7 (3.6)
	Unknown	3
Ann Arbor stage	I	31 (17.4)
	II	20 (10.3)
	III	23 (11.8)
	IV	118 (60.5)
	Unknown	1
B-symptoms	No	133 (67.9)
	Yes	63 (32.1)
Number of extranodal sites	None	38 (19.5)
	1	67 (34.4)
	>1	90 (46.2)
	Unknown	1
IPI	Low risk	61 (31.6)
	Low intermediate risk	44 (22.8)
	High intermediate risk	54 (27)
	High risk	34 (17.6)
	Unknown	3
Extranodal involvement	Graft involvement	39 (19.9)
	PCNSL	12 (6.1)
	CNS involvement, not primary	2 (1)
	Bone marrow involvement	22 (14.6)
	GI involvement	60 (30.8)
	Pulmonary involvement	51 (28)
Serum levels at diagnosis	Hemoglobin <10 g/dl	70 (35.7)
	LDH elevated	87 (44.4)
	Albumin <35 g/L	87 (29)
	Creatinine ≥1.5 mg/dl	83 (42.3)

AM, antimetabolites; CNI, calcineurin inhibitors; CNS, central nervous system; CS, corticosteroids; ECOG PS, eastern cooperative oncology group performance status; EBV(+), Epstein-Barr virus positive; EBV ISH, Epstein-Barr virus *in situ* hybridization; GI, gastro-intestinal; IPI, international prognostic index; IS, immunosuppressive therapy; LDH, lactate dehydrogenase; IQR, interquartile range; PCNSL, primary central nervous system lymphoma, PTLD, Post-transplant lymphoproliferative disorder.

The most frequent histological type was monomorphic PTLD (*n* = 162, 82.7%), with DLBCL being the most frequent subtype (*n* = 121; 74.7%). The cell of origin according to the Hans algorithm (28) was germinal center B-cell like (GCB) in 19/56 (33.9%) and non-germinal center B-cell like (non-GCB) in 37/56 (66.1%) in the posttransplant DLBCL-type (PT-DLBCL). These data were missing in 65 patients. The majority of GCB DLBCL were EBV(-) (94.7%), whereas the majority of non-GCB DLBCL were EBV(+) (78.4%).

Other subtypes of monomorphic PTLD included plasmablastic lymphoma (*n* = 14; 8.6%), plasma cell malignancies (*n* = 3; 1.9%), T-cell NHL (T-NHL) (*n* = 8; 4.9%), Burkitt lymphoma (*n* = 8; 4.9%), Burkitt-like lymphoma with 11q aberration (*n* = 4; 2.5%), EBV(+) marginal zone lymphoma (*n* = 1; 0.6%) and B-NHL, undefined (*n* = 3; 1.9%).

Median time from transplant to PTLD diagnosis was 4.3 years (IQR: 1.0-10.6), with many cases occurring late (>1 year after transplantation) (*n* = 147; 75.0%) or very late (>10 years after transplantation) (30) (*n* = 46; 23.6%).

### Treatment and Outcome

Treatment at first line consisted of reduction of immune suppression (RIS) (n = 178; 90.8%), rituximab (*n* = 120; 61.2%), chemotherapy (*n* = 41; 20.9%), surgery (*n* = 24; 12.2%), radiotherapy (*n* = 13; 6.6%), high-dose corticosteroids (*n* = 12; 6.1%) or antiviral treatment (*n* = 5; 2.6%). Ten patients (5.1%) received no treatment (7 supportive care, 3 spontaneous remissions of non-destructive PTLD). Eighty-three patients (42.3%) were treated with rituximab alone. Twenty-five patients were treated with RIS alone (12.8%) and 13 of these achieved a complete response (CR) (52%), of whom only 2 patients relapsed later on. Seventy-six patients (38.7%) in the cohort did not receive rituximab, mainly due to CD20 negativity (*n* = 26), treatment with RIS alone (*n* = 25), treatment in the pre-rituximab era (before 2000) (*n* = 19) and no treatment received (*n* = 10).

**FIGURE 3:**
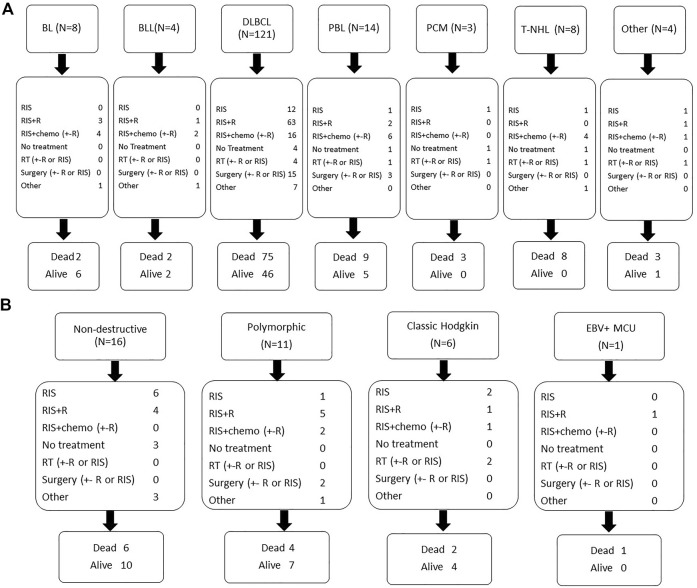
First line treatment and outcome according to histological subtype monomorphic PTLD **(A)** and other histological subtypes **(B)**. Legend: BL: Burkitt lymphoma; BLL(11q): Burkitt-like lymphoma with 11q aberration; B-NHL,u: B-cell non-Hodgkin’s lymphoma, undefined; DLBCL: diffuse large B-cell lymphoma; MCU: mucocutaneous ulcus; MZL: marginal zone lymphoma; PBL: plasmablastic lymphoma; PCM: plasma cell malignancy; PTLD: post-transplant lymphoproliferative disorder; RIS: reduction of immunosuppression; R: rituximab; RT: radiotherapy; T-NHL: T-cell non-Hodgkin’s lymphoma.

Response to first-line treatment was CR in 99 patients (50.5%), partial response in 25 (12.8%), stable disease in 9 (4.6%) and progressive disease in 40 patients (20.4%). Sixteen patients (8.2%) died during first line treatment and seven had received supportive care alone. Fifty-nine patients (30.1%) were refractory to first line treatment and 19 patients (9.7%) relapsed after achieving a CR. First line treatment according to histological subtypes is summarized in Figure 3.

**FIGURE 4:**
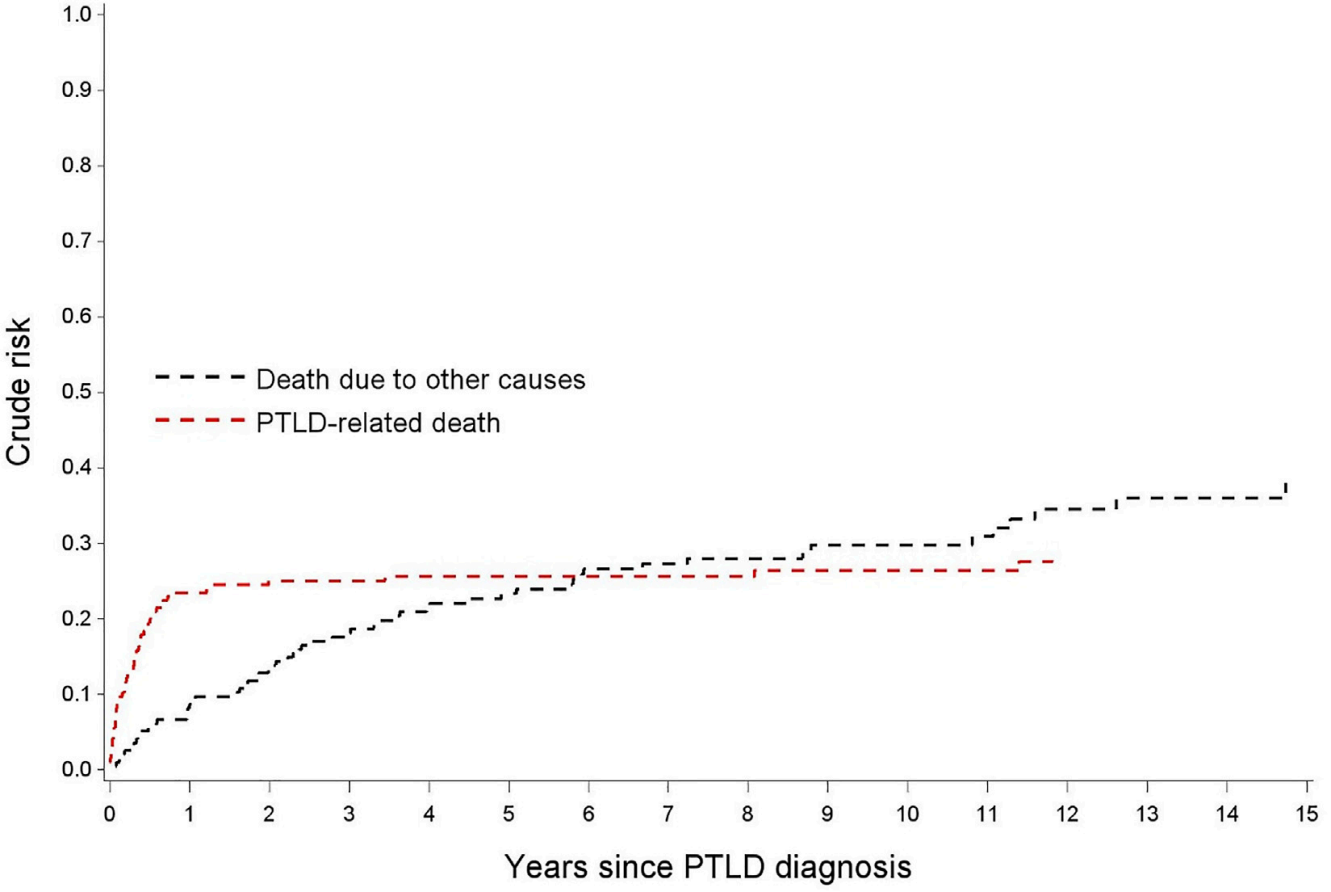
Nelson-Aalen estimates for the cumulative incidence of PTLD-related death and for death due to other causes.

After a median follow-up of 4.0 years (IQR: 0.5-8.8) after PTLD diagnosis, 115 patients (58.7%) died. Death was considered PTLD related in 46.1% (*n* = 53), non-PTLD related in 47% (*n* = 54) and unknown in 7% (*n* = 8). Other causes of death included mainly infections and other malignancies (Table 2). The cumulative incidence of PTLD-related death *versus* non-PTLD-related death is visualized in Figure 4.

**TABLE 2:** Reasons of death.

	Number (N = 115)	%
PTLD progression	47	40.9
Infections	21	18.3
Other malignancies	11	9.6
CVA	2	1.7
Bleeding	3	2.6
Cardiac events	7	6.1
MOF	5	4.3
Other	8	7
Unknown	11	9.6

CVA, cerebrovascular accident; MOF, multiple organ failure; PTLD, post-transplant lymphoproliferative disorder.

**FIGURE 5:**
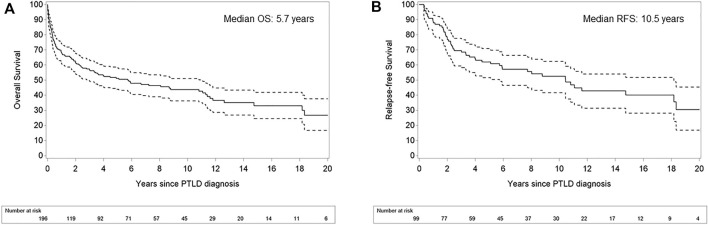
Kaplan Meier plots for overall survival in patients with post-transplant lymphoproliferative disorder **(A)** and relapse-free survival after achievement of complete response **(B)**. Legend: Dashed lines refer to the pointwise 95% confidence interval. OS: overall survival; RFS: relapse-free survival.

OS rates after PTLD for the whole cohort were 67.8, 61.7 and 51.2% after 1, 2 and 5 years, respectively. The median OS was 5.7 years (95% CI 2.99–11.07). In the 99 patients achieving a CR after first line treatment, RFS was 87.9, 77.8 and 62.0% after 1, 2 and 5 years, respectively (Figure 5).

### Uni- and Multivariate Analysis of Factors Influencing Outcome

Factors influencing CR rate in first line, PTLD-related death, OS, and RFS are summarized in Tables 3–6, respectively.

**TABLE 3:** Univariate and multivariate analysis (Logistic regressions) of factors influencing complete response rate.

	Univariate		Multivariate^a^	*p*-value
Variable	Odds Ratio (95% CI)	*p*-value	Odds Ratio (95% CI)	
Age at transplantation (years)	0.984 (0.970;1.000)	0.0430		
Age at PTLD diagnosis (years)	0.980 (0.966;0.995)	0.0096	0.989 (0.973;1.006)	0.2045
Age at PTLD diagnosis >60 years	0.437 (0.242;0.790)	0.0061		
EBV ISH positivity	1.351 (0.741;2.463)	0.3257	1.454 (0.758;2.788)	0.2596
Female gender	0.809 (0.449;1.459)	0.4818		
Transplanted organ		0.5318		
Kidney^b^	0.647 (0.280;1.496)	0.3082		
Liver^b^	0.483 (0.174;1.342)	0.1627		
Lung^b^	0.583 (0.234;1.450)	0.2458		
Graft organ involved	1.267 (0.622;2.581)	0.5145		
Monomorphic histology	0.423 (0.193;0.924)	0.0309		
CNS involvement		0.9992		
PCNSL	0.978 (0.304;3.147)	0.9706		
Secondary	0.978 (0.060;15.879)	0.9877		
Extranodal disease	0.534 (0.257;1.107)	0.0916		
Elevated LDH	0.305 (0.169;0.550)	<0.0001		
CD20 positivity		0.3238		
Positive	1.779 (0.808;3.913)	0.1524		
Partially positive	1.187 (0.225;6.260)	0.8394		
Hypoalbuminemia	0.672 (0.378;1.194)	0.1752		
IPI score	0.657 (0.528;0.817)	0.0002	0.659 (0.522;0.833)	0.0005
ECOG PS		0.0017		
ECOG 2^c^	0.560 (0.279;1.126)	0.1036		
ECOG 3/4^c^	0.115 (0.025;0.529)	0.0055		
Ann Arbor stage III-IV	0.451 (0.236;0.864)	0.0163		
Year of PTLD diagnosis	0.961 (0.922;1.003)	0.0661	0.955 (0.913;0.999)	0.0436

^a^ EBV status was added into the multivariate model obtained after backward selection

^b^ Compared to heart transplant.

^c^ Compared to ECOG PS 0-1.

95% CI, 95% confidence interval; PTLD, post-transplant lymphoproliferative disorder; ECOG PS, eastern cooperative oncology group performance status; EBV ISH, Epstein-Barr Virus *in situ* hybridization; LDH, lactate dehydrogenase; IPI, international prognostic index; PCNSL, primary central nervous system lymphoma.

**TABLE 4:** Univariate and multivariate analysis (Cox regressions) of patients characteristics related to PTLD related death.

	Univariate		Multivariate^a^	
Variable	Hazard Ratio (95% CI)	*p*-value	Hazard Ratio (95% CI)	*p*-value
Age at transplantation (years)	1.029 (1.012;1.045)	0.0007		
Age at PTLD diagnosis (years)	1.030 (1.013;1.047)	0.0006		
Age at PTLD diagnosis >60 years	2.798 (1.617;4.842)	0.0002		
EBV ISH positivity	1.670 (0.884;3.157)	0.1143	1.155 (0.591;2.255)	0.6730
Female gender	0.860 (0.478;1.549)	0.6161		
Transplanted organ		0.9320		0.0162
Kidney^b^	0.855 (0.392;1.867)	0.6945	1.124 (0.498;2.534)	0.7787
Liver^b^	1.068 (0.424;2.694)	0.8883	2.477 (0.924;6.639)	0.0714
Lung^b^	1.013 (0.438;2.342)	0.9762	4.074 (1.456;11.399)	0.0075
Graft organ involved	0.834 (0.407;1.710)	0.6207	0.322 (0.135;0.772)	0.0111
Monomorphic histology	3.365 (1.211;9.352)	0.0200		
CNS involvement		0.2785		
PCNSL	2.021 (0.802;5.094)	0.1359		
Secondary	2.820 (0.388;20.494)	0.3055		
Extranodal disease	2.782 (1.105;7.003)	0.0298		
Elevated LDH	5.274 (2.799;9.937)	<0.0001		
CD20 positivity		0.3068		
Positive	0.587 (0.307;1.122)	0.1073		
Partially positive	0.708 (0.158;3.166)	0.6510		
Hypoalbuminemia	3.566 (1.939;6.561)	<0.0001	2.398 (1.256;4.577)	0.0080
IPI score	1.935 (1.562;2.399)	<0.0001	1.978 (1.554;2.519)	<0.0001
ECOG PS		<0.0001		
ECOG 2^c^	2.196 (1.163;4.148)	0.0153		
ECOG 3/4^c^	9.207 (4.581;18.504)	<0.0001		
Ann Arbor stage III-IV	4.306 (1.711;10.836)	0.0019		
Year of PTLD diagnosis	0.951 (0.916;0.988)	0.0100	0.937 (0.897;0.979)	0.0038

^a^ EBV status was added into the multivariate model obtained after backward selection.

^b^ Compared to heart transplant.

^c^ Compared to ECOG PS 0-1.

Abbreviations: 95% CI: 95% confidence interval; PTLD: post-transplant lymphoproliferative disorder; ECOG PS: eastern cooperative oncology group performance status; EBV ISH: Epstein-Barr Virus *in situ* hybridization; LDH: lactate dehydrogenase; IPI: international prognostic index; PCNSL: primary central nervous system lymphoma.

**TABLE 5:** Univariate and multivariate analysis (Cox regressions) of patient characteristics related to overall survival.

	Univariate		Multivariate^a^	
Variable	Hazard Ratio (95% CI)	*p*-value	Hazard Ratio (95% CI)	*p*-value
Age at transplantation (years)	1.040 (1.028;1.052)	<0.0001		
Age at PTLD diagnosis (years)	1.041 (1.028;1.053)	<0.0001	1.035 (1.022;1.049)	<0.0001
Age at PTLD diagnosis >60 years	3.389 (2.321;4.948)	<0.0001		
EBV ISH positivity	1.475 (0.975;2.232)	0.0659	1.441 (0.928;2.239)	0.1037
Female gender	0.998 (0.670;1.484)	0.9903	1.290 (0.837;1.986)	0.2483
Transplanted organ		0.6780		0.0161
Kidney^b^	0.854 (0.506;1.442)	0.5553	1.197 (0.685;2.093)	0.5282
Liver^b^	1.186 (0.637;2.209)	0.5912	2.291 (1.181;4.445)	0.0142
Lung^b^	0.972 (0.546;1.729)	0.9226	2.091 (1.084;4.033)	0.0278
Graft organ involved	1.091 (0.690;1.725)	0.7088		
Monomorphic histology	2.468 (1.381;4.409)	0.0023		
CNS involvement		0.6513		
PCNSL	1.422 (0.691;2.925)	0.3393		
Secondary	1.224 (0.171;8.792)	0.8405		
Extranodal disease	1.879 (1.121;3.151)	0.0167		
Elevated LDH	2.922 (1.997;4.275)	<0.0001		
CD20 positivity		0.2877		
Positive	0.751 (0.466;1.210)	0.2393		
Partially positive	0.394 (0.092;1.683)	0.2085		
Hypoalbuminemia	2.758 (1.873;4.062)	<0.0001	1.956 (1.289;2.967)	0.0016
IPI score	1.612 (1.399;1.856)	<0.0001	1.346 (1.154;1.570)	0.0002
ECOG PS		<0.0001		
ECOG 2^c^	1.715 (1.127;2.608)	0.0117		
ECOG 3/4^c^	4.815 (2.636;8.795)	<0.0001		
Ann Arbor stage III-IV	1.902 (1.211;2.989)	0.0053		
Year of PTLD diagnosis	0.968 (0.942;0.995)	0.0196	0.962 (0.931;0.993)	0.0172

^a^ Year of PTLD diagnosis was added into the multivariate model obtained after backward selection

^b^ Compared to heart transplant.

^c^ Compared to ECOG PS 0-1.

95% CI, 95% confidence interval; PTLD, post-transplant lymphoproliferative disorder; ECOG PS, eastern cooperative oncology group performance status; EBV ISH, Epstein-Barr Virus *in situ* hybridization; LDH, lactate dehydrogenase; IPI, international prognostic index; PCNSL, primary central nervous system lymphoma.

**TABLE 6:** Univariate and multivariate analysis (Cox regressions) of patient characteristics related to relapse free survival.

	Univariate		Multivariate	
Variable	Hazard Ratio (95% CI)	*p*-value	Hazard Ratio (95% CI)	*p*-value
Age at transplantation (years)	1.039 (1.022;1.057)	<0.0001		
Age at PTLD diagnosis (years)	1.047 (1.029;1.066)	<0.0001	1.054 (1.034;1.074)	<0.0001
Age at PTLD diagnosis >60 years	3.576 (2.047;6.247)	<0.0001		
EBV ISH positivity	1.261 (0.678;2.346)	0.4647	2.183 (1.075;4.432)	0.0307
Female gender	0.989 (0.541;1.810)	0.9726		
Transplanted organ		0.2155		0.0103
Kidney^a^	1.000 (0.486;2.056)	0.9993	1.585 (0.734;3.424)	0.2414
Liver^a^	1.782 (0.736;4.313)	0.2003	5.244 (1.904;14.446)	0.0013
Lung^a^	0.645 (0.266;1.561)	0.3306	1.398 (0.510;3.831)	0.5153
Graft organ involved	0.903 (0.453;1.801)	0.7726		
Monomorphic histology	1.519 (0.759;3.041)	0.2376		
CNS involvement		0.5534		
PCNSL	0.862 (0.267;2.782)	0.8044		
Secondary	ND	0.9884		
Extranodal disease	1.352 (0.694;2.631)	0.3751		
Elevated LDH	2.200 (1.248;3.879)	0.0064		
CD20 positivity		0.1585		
Positive	1.317 (0.522;3.319)	0.5598		
Partially positive	ND	0.9873		
Hypoalbuminemia	2.371 (1.354;4.152)	0.0025		
IPI score	1.417 (1.146;1.751)	0.0013		
ECOG PS		0.0417		
ECOG 2^b^	1.924 (1.054;3.515)	0.0332		
ECOG 3/4^b^	ND	0.9897		
Ann Arbor stage III-IV	1.143 (0.644;2.027)	0.6479		
Year of PTLD diagnosis	0.969 (0.931;1.009)	0.1280	0.975 (0.929;1.024)	0.3078

^a^ Compared to heart transplant.

^b^ Compared to ECOG PS 0-1.

95% CI, 95% confidence interval; PTLD, post-transplant lymphoproliferative disorder; ECOG PS, eastern cooperative oncology group performance status; EBV ISH, Epstein-Barr Virus *in situ* hybridization; LDH, lactate dehydrogenase; IPI, international prognostic index; PCNSL, primary central nervous system lymphoma; ND, not determined.

Higher age at transplantation, higher age at PTLD diagnosis, monomorphic histology, elevated LDH, higher IPI, poor ECOG PS (3,4) and advanced Ann Arbor stage were statistically significant adverse factors for CR rate in univariate analysis. In multivariate analysis a higher IPI score and a higher year of PTLD diagnosis were related to a lower CR rate.

Higher age at transplantation, higher age at PTLD diagnosis, monomorphic histology, extranodal disease, elevated LDH, hypoalbuminemia, higher IPI, poor ECOG PS (>1), advanced Ann Arbor stage are significantly related to PTLD-related death in univariate analysis using Cox regression models. Similar results were obtained using Fine and Gray models (results not shown). In the multivariate model hypoalbuminemia, higher IPI-score, graft organ involvement and type of transplanted organ (lung *versus* heart) were retained as factors associated with worse outcome. A higher year of PTLD diagnosis was associated with less PTLD-related death in uni- and multivariate analysis.

Higher age at transplantation, higher age at PTLD diagnosis, monomorphic histology, extranodal disease, elevated LDH, hypoalbuminemia, a higher IPI-score, ECOG >1, advanced Ann Arbor stage were significantly adverse factors for OS in univariate analysis. In the multivariate model the IPI-score, higher age at diagnosis, hypoalbuminemia, type of transplanted organ (liver and lung transplantation compared to heart) were retained as poor prognostic factors. Higher year of PTLD diagnosis was associated with a longer OS in uni- and multivariate analysis.

Higher age at transplantation, higher age at PTLD diagnosis, elevated LDH, hypoalbuminemia, higher IPI, poor ECOG PS were significant adverse factors for RFS in univariate analysis. In the multivariate model higher age at diagnosis, EBV positivity and liver transplantation were considered prognostic factors worse RFS.

In summary, IPI was an important prognostic factor, significantly related to all four outcomes in univariate analysis and to CR rate, PTLD-related death and OS in multivariate analysis. Furthermore, hypoalbuminemia was a poor prognostic factor for PTLD-related death, OS and RFS in univariate analysis and for PTLD-related death and OS in multivariate analysis. Type of transplanted organ was significantly related to RFS, PTLD-related death and OS in multivariate analysis.

### EBV

EBV status, as determined by EBV ISH at the time of diagnosis, was positive in 119 of the 186 evaluable cases (64%). The number of positive EBV was higher in early (<1 year after transplantation) PTLD cases (*n* = 43; 89.6%) compared to late PTLD (*n* = 76; 55.1%). EBV positivity was associated with type of grafted organ (highest in lung, lowest in liver transplantation) and organ-involvement in the whole PTLD cohort. There was no association between EBV status and other clinical factors (Table 7).

**TABLE 7:** Comparison of baseline characteristics in relation to EBV status.

	EBV negative (N = 67)	EBV positive (N = 119)	p
Male Gender	45 (67.2%)	76 (63.2%)	0.75
Transplanted organ			
Heart	8 (12%)	21 (17.7%)	0.02
Liver	14 (20.1%)	13 (10.9%)	
Lung	11 (16.4%)	39 (32.8%)	
Kidney	34 (50.8%)	46 (38.7%)	
Graft organ involvement	7 (10.5%)	29 (24.4%)	0.021
Monomorphic PTLD	54 (80.6%)	98 (82.4%)	0.84
CNS involvement	2 (3%)	12 (10.1%)	0.27
CD20 positive	52 (78.8%)	96 (82.1%)	0.27
Decreased albumin	26 (38.5%)	57 (50%)	0.16
Median age at PTLD (years)	56	52.6	0.18
Median IPI	2	2	0.37
Initial therapy			0.090
RIS alone	5 (7.5%)	17 (14.3%)	
RIS + other (excluding R/chemo)	5 (7.5%)	13 (10.9%)	
RIS + R	40 (59.7%)	54 (45.4%)	
RIS + chemo	10 (14.9%)	9 (7.6%)	
RIS + R + chemo	3 (4.5%)	15 (12.6%)	
Other	4 (6.0%)	11 (9.2%)	

chemo, chemotherapy; EBV, Epstein-Barr Virus; IPI, international prognostic index; CNS, central nervous system; PTLD, Post-transplant lymphoproliferative disorder, R, rituximab; RIS, reduction of immunosuppression.

**FIGURE 6:**
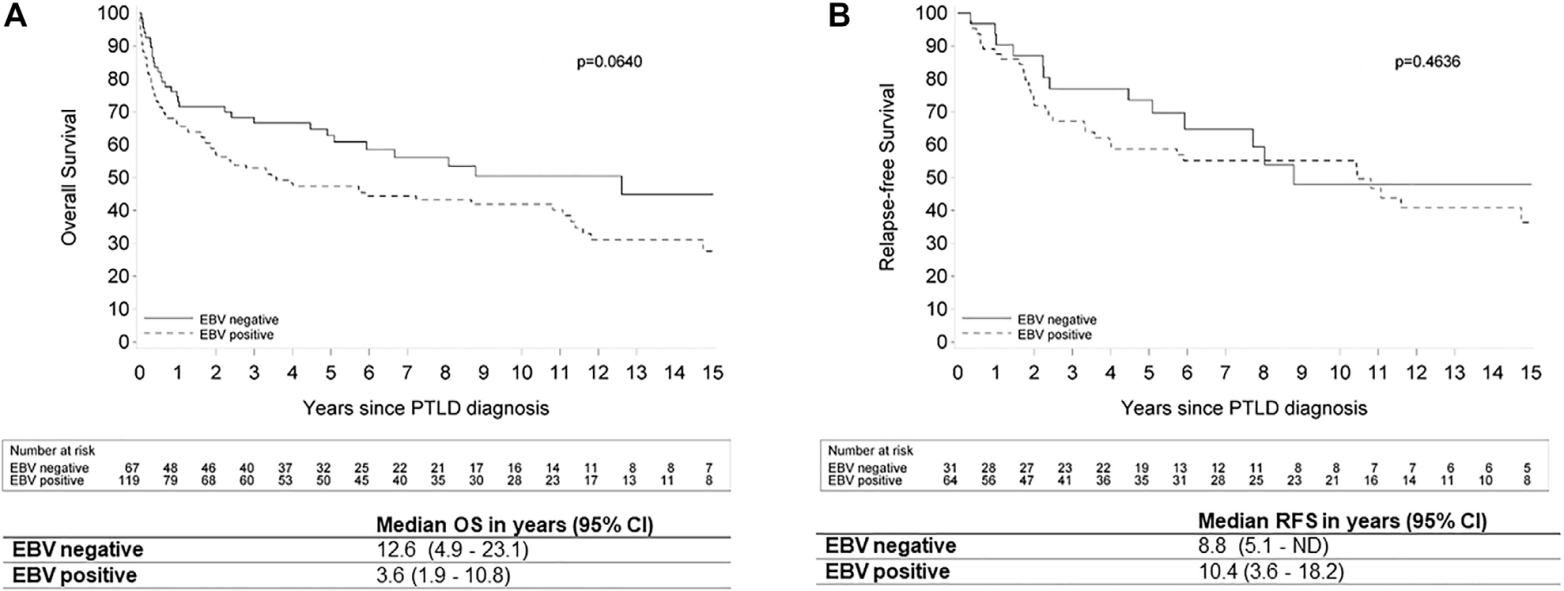
Kaplan Meier plots for **(A)** overall survival and **(B)** relapse-free survival by EBV status in patients with post-transplant lymphoproliferative disorder. Legend: EBV: Epstein-Barr Virus; OS: overall survival; ND: not determined; PTLD: post-transplant lymphoproliferative disorder; RFS: relapse-free survival; 95% CI: 95% confidence interval.

EBV status at diagnosis was not significantly related to OS in univariate (hazard ratio (HR): 1.48 (95% CI: 0.975–2.232); *p* = 0.066) and multivariate analysis (HR: 1.44 (95% CI: 0.928–2.239); *p* = 0.10). However, there was a trend towards worse OS for the EBV(+) PTLD. There was also no significant relation between EBV status and CR (odds ratio (OR): 1.35 (95% CI: 0.741–2.463); *p* = 0.33) and PTLD-related death (HR: 1.67 (95% CI: 0.884–3.157); *p* = 0.11) in univariate, nor in multivariate analysis ((OR: 1.45 (95% CI: 0.758–2.788); *p* = 0.26) and (HR: 1.15 (0.591–2.255); *p* = 0.67), respectively). However, there was a relation between EBV status and RFS in the multivariate model, where EBV positivity was a risk factor (HR: 2.29 (95% CI: 1.146–4.595); *p* = 0.02) (Figure 6).

**FIGURE 7:**
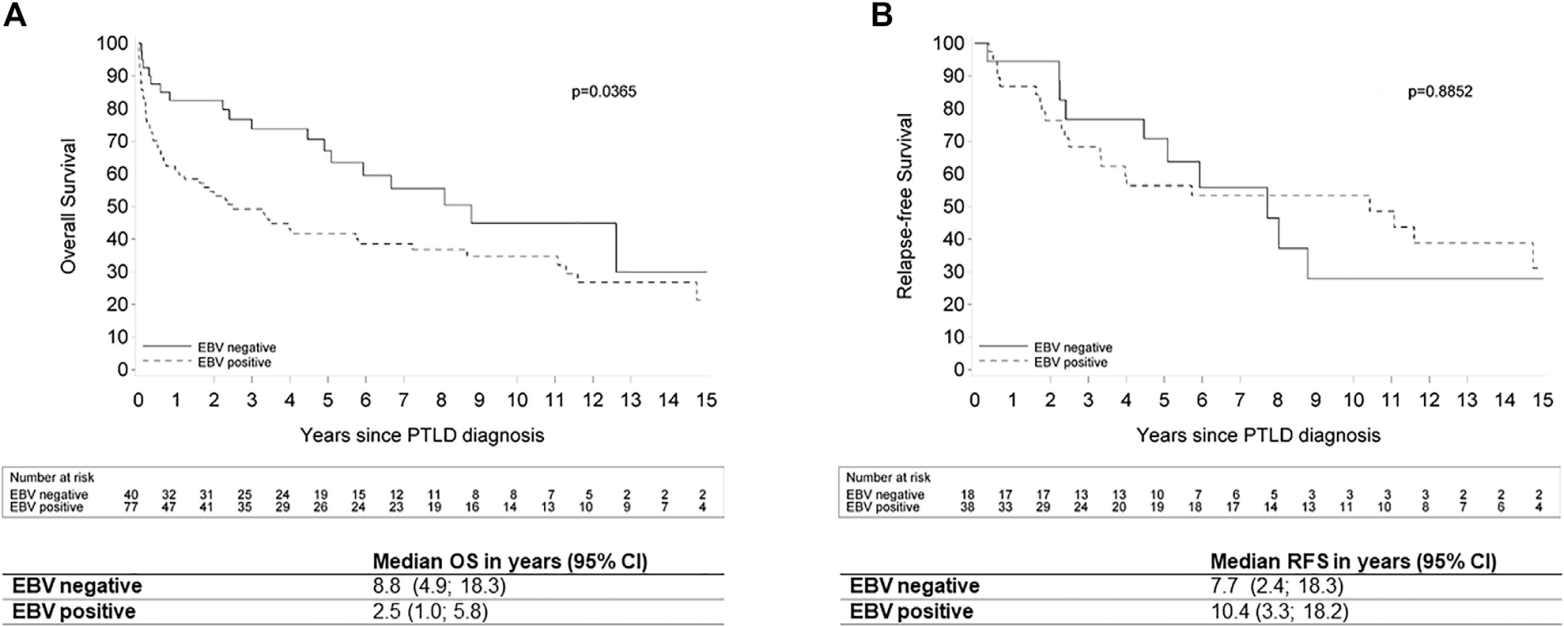
Kaplan Meier plots for **(A)** overall survival and **(B)** relapse-free survival by EBV status in patients with post-transplant diffuse large B-cell lymphoma. Legend: EBV: Epstein-Barr Virus; OS: overall survival; ND: not determined; PTLD: post-transplant lymphoproliferative disorder; RFS: relapse-free survival; 95% CI: 95% confidence interval.

A subgroup analysis of all cases of PT-DLBCL showed that EBV ISH was positive in 77 of the 117 evaluable cases (65.8%). Furthermore, we saw a significantly better median OS for EBV(−) PT-DLBCL compared to EBV(+) PT-DLBCL (8.8 versus 2.5 years respectively; *p* = 0.0365).There was no significant relation between EBV status and RFS in this group (*p* = 0.8852) (Figure 7).

EBV PCR in blood was positive in 107 of 142 evaluable cases (75.4%). However, EBV PCR was more often positive in EBV ISH positive cases (91% of 89 evaluable cases), than in EBV ISH negative cases (52% of 50 evaluable cases). This resulted in a sensitivity of 91% and specificity of 48% for EBV PCR in predicting EBV ISH positivity.

### Era of PTLD Diagnosis

There was a significant relation between year of PTLD diagnosis and OS, that persisted after correction for differences in patient mix in the multivariate model: the more recent the PTLD diagnosis, the lower the risk for death (HR: 0.97 (95% CI: 0.942–0.995; *p* = 0.0196) and adjusted HR: 0.962 (95%CI: 0.931–0.933; *p* = 0.017) in the Cox multivariate model.

A similar result was obtained for PTLD-related death: HR: 0.951 (95% CI: 0.916–0.988; *p* = 0.01) and adjusted HR: 0.935 (95% CI: 0.896–0.977; *p* = 0.0024) for the year of PTLD diagnosis in the multivariate Cox model. A similar conclusion was obtained in the Fine and Gray model (results not shown). However, there was no evidence of a significant relation between year of PTLD diagnosis and CR or RFS.

## Discussion

We investigated the baseline characteristics, outcome, role of EBV and era of PTLD diagnosis on outcome in a large cohort of biopsy-proven PTLD after SOT. We noticed a high proportion of late (>1 year after transplantation: *n* = 147; 75%) and very late PTLD (>10 years after transplantation; *n* = 46; 23.6%) in our analysis. Several reports have recently suggested that the incidence of early EBV(+) PTLD is decreasing (3, 11, 31). In our cohort the proportion of early PTLD was stable over the first, second and third decade (21.1%, 20.9% and 28.1% respectively). Other groups have suggested that a decrease in early PTLD might be a result of pre-emptive EBV viral load monitoring. However, this has not been confirmed in a recent report (11) and this strategy has not been implemented in our series. Other factors influencing the incidence of early PTLD include the changes in immunosuppressive regimens and decreased use of T-cell depleting induction therapy (32-35).

The median age at diagnosis in the current study was 54.1 years, which is comparable to previous reports (26,36-38). PTLD is typically diagnosed at an advanced stage (72.3%) with extra-nodal involvement (80.5%). Gastro-intestinal involvement (30.8%) was the most frequent extra-nodal site involved. We observed 12 cases of PCNSL (6.1%) in our cohort, less than the previously reported 10% of all PTLDs (39-41). However, it is difficult to draw definite conclusions regarding the incidence of PCNSL in PTLD due to the small group size. By far the most commonly observed histologic type of PTLD in our study was monomorphic PTLD (82.7%), with DLBCL as the most frequent subtype. Non-destructive and classic Hodgkin lymphoma PTLD were rare, as previously reported in the literature. Furthermore, we noted only 11 cases (5.6%) of polymorphic PTLD, which is less than previously reported (3, 26, 37, 42). A more recent report noted a similar rate, with 5.7% polymorphic PTLD in a single center analysis of 227 adult PTLD after SOT (14). Tsai et al. also reported that PTLD morphology has changed over the past 3 decades, with a gradual increase in the number of monomorphic PTLD and a steady number of polymorphic PTLD (38). This seems to be corroborated by our results.

Burkitt lymphoma type PTLD is a rare entity, with only 8 cases over 30 years in our study. However, their prognosis is relatively good as 6 patients are currently alive and still in remission after treatment with intensified immuno/chemotherapy. We encountered 4 cases of Burkitt-like lymphoma with 11q aberration, a rare entity known to be more prevalent in immunocompromised patients (43). Furthermore, we encountered 8 T-NHLs, of which 2 were classified as hepatosplenic T-cell lymphoma and 3 cases were primary cutaneous T-NHL. Prognosis was very poor in these patients with 6 of them dying within 1 year after the diagnosis. The poor prognosis of T-cell PTLD has previously been reported (29, 44-48). A more recent report by Barba et al showed that the outcome in 58 T/NK-cell PTLD after kidney transplantation was worse than in 148 T/NK-cell lymphomas in non-transplanted (49). They noted that transplant recipients received less anthracycline-based therapy, probably out of fear of complications in this fragile population. EBV(+) mucocutaneous ulcer has recently been described as an indolent entity occurring in patients with age-related or iatrogenic immunosuppression (2). It is currently classified as a separate entity (outside PTLD) in the WHO 2017 classification (2). However, it can occur in the post-transplant setting and needs to be considered in the differential diagnosis. We reclassified only one case of EBV(+) mucocuteanous ulcer in our cohort, which was originally classified as monomorphic PTLD, DLBCL type.

Most cases of PTLD are related to EBV. However, more recent reports suggest that up to 50% of PTLDs are EBV(−) (50). In our cohort EBV ISH was positive in 64% of all evaluable cases. Analysis of EBV DNA viremia showed a high sensitivity (91%), but low specificity (48%) in predicting EBV ISH status. Previous studies have shown that transplant recipients with PTLD have a higher viral load then recipients without PTLD. Furthermore, a higher or rapidly increasing viral load is associated with a higher risk of PTLD (4, 51-54). The low specificity of the EBV PCR in our series could possibly be attributed to the low cut-off value used (>2.7 log copies/ml or >2.18 log EBV IU/ml).

Genomic and transcriptional studies have recently demonstrated that EBV(+) and EBV(−) PTLD carry different genomic signatures(16,17). The genomic aberrations in EBV(−) PTLD are less complex and indistinguishable from those in immunocompetent DLBCL. This has led to the hypothesis that EBV(+) PT-DLBCL represent true PTLD and that EBV(−) PT-DLBCL could be considered as *de novo* lymphomas in transplant recipients (16,17). EBV(+) and EBV(-) PT-DLBCL have some different clinical characteristics. In particular, EBV(+) PT-DLBCL typically occurs early and is most often non-GCB type, whereas EBV(−) PT-DLBCL occurs later and is typically of GCB type. Furthermore, polymorphic or non-destructive lesions are usually EBV(+)(4, 16, 55). Despite these differences both groups are essentially treated with the same therapy (except EBV-specific adoptive immunotherapy). The impact of EBV status on treatment response or prognosis remains unclear (50, 56). In our cohort we found no significant relation between EBV status and CR, PTLD-related death or OS. However, we observed a significant relation between EBV status and OS in PT-DLBCL, with clinically meaningful improved survival in EBV(-) PT-DLBCL compared to EBV(+) PT-DLBCL (8.8 years versus 2.5 years, respectively). Previous reports have shown conflicting results on the relation between EBV status and OS (13, 14, 18, 25, 50, 57, 58).

As only 21 patients were treated before 2000 (when rituximab became available in Belgium), no comparison could be made regarding outcomes in the pre- and post-rituximab era. However, we investigated the impact of date of PTLD diagnosis on outco-me parameters. We observed a significant improvement in OS and a diminished PTLD-related death rate with later year of PTLD diagnosis. This relation was not found with CR and RFS. It seems that the prognosis of PTLD has improved over the past decades, although the responses to first line treatment have not. Possible explanations for this finding could be achievement of deeper responses, better supportive care and risk-stratified sequential therapy (patients not achieving CR to rituximab monotherapy can still be rescued with R-CHOP chemotherapy).

RIS remains the cornerstone of PTLD treatment. Twenty-five patients were treated with RIS alone and 13 of these achieved a CR (52%). Reported response rates to RIS have been very variable, however the largest earlier reported single-center retrospective analysis of 67 PTLDs after SOT treated with RIS alone, reported an overall response rate of 45% (37% CR) (59). Responses have been known to be higher in non-destructive lesions and in EBV(+) PTLD (4). The higher rate of responses in our cohort might reflect the higher ratio of non-destructive and polymorphic lesions. Of note, RIS may be related to subsequent onset of (chronic) rejection, for instance in lung transplant recipients, which requires increased clinical surveillance (60).

The median OS in our cohort was 5.7 years. This is less than reported in the prospective phase II PTLD-1 and PTLD-2 trials, with a median OS of 6.6 years (24,25). However, only CD20-positive PTLD were included in these PTLD-1 and 2 trials. More recent real-world data showed a 3 years OS of 65.9% in CD20-positive PTLD treated with rituximab-based therapy (61). The IPI-score remained the most important poor prognostic factor in multivariate analysis for OS, CR and PTLD-related death in the current study, in concordance with earlier reports. Hypoalbuminemia and type of organ transplanted (liver and lung) were also retained in our multivariate model as poor prognostic factors for OS.

This study is limited by its retrospective design. Treatment of PTLD has obviously changed over the past decades with the incorporation of rituximab into first line treatment of CD20-positive PTLD since the early 2000s. Furthermore, some data regarding EBV serology and EBV PCR in blood were missing, since this only came into practice in the last 2 decades. Some patients reported in the current study were also reported in a previous publication (18). However, the latter study also included PLTD after HSCT and the follow-up was shorter than in the current study. In addition, we reclassified all PTLD according to the WHO 2017 classificiation (2) and added more detailed histopathological data (such as cell of origin).

In conclusion, this retrospective analysis provides real world data on 196 biopsy-proven PTLD cases, to the best of our knowledge the second largest single-institution cohort published in the literature. The OS of our patients increased in the past decade, resulting in a median OS of 5.7 years for the whole cohort. We observed a significantly improved OS for EBV(−) PT-DLBCL compared to EBV(+) PT-DLBCL.

## Data Availability

Data concerns health-related information of the patients and therefore cannot be given away freely. If needed, the first author can be contacted to obtain the data.

## Glossary

[^18^F]FDG-PET/CT,: Positron emission tomography with ^18^F-fluorodeoxyglucose combined with computed tomography
ATG,: anti-thymocyte globulin
B-NHL,: B-cell non-Hodgkin lymphoma
CI,: Confidence Interval
CNS,: Central nervous system
CR,: complete response
CT,: computed tomography
DLBCL,: diffuse large B-cell lymphoma
EBER,: Epstein Barr-encoded RNA
EBV,: Epstein Barr Virus
EBV(+),: Epstein Barr Virus positive
EBV(-),: Epstein Barr Virus negative
EBV ISH,: Epstein Barr Virus *in situ* hybridization
ECOG PS,: Eastern Cooperative Oncology Group Performance status
GCB,: germinal center B-cell like
GI,: gastro-intestinal
HR,: hazard ratio
HSCT,: hematopoietic stem cell transplantation
IPI,: International Prognostic Index
IQR,: interquartile range
LDH,: lactate dehydrogenase
NHL,: non-Hodgkin lymphoma
OR,: Odds Ratio
OS,: overall survival
PCNSL,: primary central nervous system lymphoma
PCR,: polymerase chain reaction
PTLD,: Post-transplant lymphoproliferative disorder
PT-DLBCL,: Post-transplant diffuse large B-cell lymphoma
R-CHOP,: rituximab, cyclophosphamide, doxorubicine, vincristine and prednisolone
RFS,: relapse-free survival
RIS,: reduction of immune suppression
SOT,: solid organ transplantation
T-NHL,: T-cell non-Hodgkin lymphoma
WHO,: World Health Organization

## References

[1] Penn I. Cancers Complicating Organ Transplantation. N Engl J Med (1990) 323:1767–9. 10.1056/NEJM199012203232510 2247108

[2] Swerdlow SH Campo E Harris NL. WHO Classification of Tumours of Haematopoietic and Lymphoid Tissues. 4th ed. Lyon: IARC (2017). p. 453–62.

[3] Caillard S Lamy FX Quelen C Dantal J Lebranchu Y Lang P Epidemiology of Posttransplant Lymphoproliferative Disorders in Adult Kidney and Kidney Pancreas Recipients: Report of the French Registry and Analysis of Subgroups of Lymphomas. Am J Transpl (2012) 12(3):682–93. 10.1111/j.1600-6143.2011.03896.x 22226336

[4] Dierickx D Habermann TM. Post-Transplantation Lymphoproliferative Disorders in Adults. N Engl J Med (2018) 378(6):549–62. 10.1056/NEJMra1702693 29414277

[5] Dharnidharka VR. Comprehensive Review of post–organ Transplant Hematologic Cancers. Am J Transpl (2018) 18(3):537–49. 10.1111/ajt.14603 29178667

[6] Sampaio MS Cho YW Qazi Y Bunnapradist S Hutchinson IV Shah T. Posttransplant Malignancies in Solid Organ Adult Recipients: an Analysis of the U.S. National Transplant Database. Transplantation (2012) 94:990–8. 10.1097/TP.0b013e318270bc7b 23085553

[7] Allen UD Preiksaitis JK. Post-Transplant Lymphoproliferative Disorders, Epstein-Barr Virus Infection, and Disease in Solid Organ Transplantation: Guidelines from the American Society of Transplantation Infectious Diseases Community of Practice. Clin Transpl (2019) 33(9):e13652–22. 10.1111/ctr.13652 31230381

[8] Shannon-Lowe C Rickinson AB Bell AI. Epstein-barr Virus-Associated Lymphomas. Philos Trans R Soc Lond B Biol Sci (2017) 372(1732):20160271. 10.1098/rstb.2016.0271 28893938 PMC5597738

[9] Green M Michaels MG. Epstein-barr Virus Infection and Posttransplant Lymphoproliferative Disorder. Am J Transpl (2013) 13(3):41–54. 10.1111/ajt.12004 23347213

[10] Morscio J Tousseyn T. Recent Insights in the Pathogenesis of post-transplantation Lymphoproliferative Disorders. World J Transpl (2016) 6(3):505–16. 10.5500/wjt.v6.i3.505 PMC5036120 27683629

[11] Peters AC Akinwumi MS Cervera C Mabilangan C Ghosh S Lai R The Changing Epidemiology of Posttransplant Lymphoproliferative Disorder in Adult Solid Organ Transplant Recipients over 30 Years: A Single-center Experience. Transplantation (2018) 102(9):1553–62. 10.1097/TP.0000000000002146 29485513

[12] Maksten EF Vase MØ Kampmann J D’Amore F Møller MB Strandhave C Post-Transplant Lymphoproliferative Disorder Following Kidney Transplantation: A Population-Based Cohort Study. Transpl Int (2016) 29(4):483–93. 10.1111/tri.12744 26749337

[13] Evens AM David KA Helenowski I Nelson B Kaufman D Kircher SM Multicenter Analysis of 80 Solid Organ Transplantation Recipients with Post-Transplantation Lymphoproliferative Disease: Outcomes and Prognostic Factors in the Modern Era. J Clin Oncol (2010) 28:1038–46. 10.1200/JCO.2009.25.4961 20085936 PMC2834429

[14] King RL Khurana A Mwangi R Fama A Ristow KM Maurer MJ Clinicopathologic Characteristics, Treatment, and Outcomes of Post-transplant Lymphoproliferative Disorders: A Single-Institution Experience Using 2017 WHO Diagnostic Criteria. Hemasphere (2021) 5(10):e640. 10.1097/HS9.0000000000000640 34514344 PMC8423401

[15] Zaffiri L Long A Neely ML Cherikh WS Chambers DC Snyder LD. Incidence and Outcome of post-transplant Lymphoproliferative Disorders in Lung Transplant Patients: Analysis of ISHLT Registry. J Hear Lung Transpl (2020). 10.1016/j.healun.2020.06.010 PMC7530100 32654913

[16] Morscio J Dierickx D Ferreiro JF Herreman A Van Loo P Bittoun E Gene Expression Profiling Reveals clear Differences between EBV-Positive and EBV-Negative Posttransplant Lymphoproliferative Disorders. Am J Transpl (2013) 13(5):1305–16. 10.1111/ajt.12196 23489474

[17] Finalet Ferreiro J Morscio J Dierickx D Vandenberghe P Gheysens O Verhoef G EBV-Positive and EBV-Negative Posttransplant Diffuse Large B Cell Lymphomas Have Distinct Genomic and Transcriptomic Features. Am J Transpl (2016) 16(2):414–25. Available from: https://pubmed.ncbi.nlm.nih.gov/26780579/ . 10.1111/ajt.13558 26780579

[18] Dierickx D Tousseyn T Sagaert X Fieuws S Wlodarska I Morscio J Single-center Analysis of Biopsy-Confirmed Posttransplant Lymphoproliferative Disorder: Incidence, Clinicopathological Characteristics and Prognostic Factors. Leuk Lymphoma (2013) 54(11):2433–40. 10.3109/10428194.2013.780655 23442063

[19] Rosenberg SA. Validity of the Ann Arbor Staging Classification for the Non-hodgkin’s Lymphomas. Cancer Treat Rep (1977) 61(6):1023–7. 902260

[20] Association WM. World Medical Association Declaration of Helsinki: Ethical Principles for Medical Research Involving Human Subjects. JAMA (2013) 310(20):2191–4. 10.1001/jama.2013.281053 24141714

[21] A predictive model for aggressive non-Hodgkin’s lymphoma. A Predictive Model for Aggressive Non-hodgkin's Lymphoma. N Engl J Med (1993) 329(14):987–94. 10.1056/NEJM199309303291402 8141877

[22] Barrington SF Mikhaeel NG Kostakoglu L Meignan M Hutchings M Müeller SP Role of Imaging in the Staging and Response Assessment of Lymphoma: Consensus of the International Conference on Malignant Lymphomas Imaging Working Group. J Clin Oncol (2014) 32(27):3048–58. 10.1200/JCO.2013.53.5229 25113771 PMC5015423

[23] Meignan M Gallamini A Haioun C Haioun C. Report on the First International Workshop on Interim-PET Scan in Lymphoma. Leuk Lymphoma (2009) 50(8):1257–60. 10.1080/10428190903040048 19544140

[24] Trappe RU Dierickx D Zimmermann H Morschhauser F Mollee P Zaucha JM Response to Rituximab Induction Is a Predictive Marker in B-Cell post-transplant Lymphoproliferative Disorder and Allows Successful Stratification into Rituximab or R-Chop Consolidation in an International, Prospective, Multicenter Phase II Trial. J Clin Oncol (2017) 35(5):536–43. 10.1200/JCO.2016.69.3564 27992268

[25] Trappe R Oertel S Leblond V Mollee P Sender M Reinke P Sequential Treatment with Rituximab Followed by CHOP Chemotherapy in Adult B-Cell post-transplant Lymphoproliferative Disorder (PTLD): The Prospective International Multicentre Phase 2 PTLD-1 Trial. Lancet Oncol (2012) 13(2):196–206. 10.1016/S1470-2045(11)70300-X 22173060

[26] Caillard S Porcher R Provot F Dantal J Choquet S Durrbach A Post-Transplantation Lymphoproliferative Disorder after Kidney Transplantation: Report of a Nationwide French Registry and the Development of a New Prognostic Score. J Clin Oncol (2013) 31(10):1302–9. 10.1200/JCO.2012.43.2344 23423742

[27] Engels EA Pfeiffer RM Fraumeni JF Kasiske BL Israni AK Snyder JJ Spectrum of Cancer Risk Among US Solid Organ Transplant Recipients. JAMA - J Am Med Assoc (2011) 30617:1891–901. 10.1001/jama.2011.1592 PMC3310893 22045767

[28] Hans CP Weisenburger DD Greiner TC Gascoyne RD Delabie J Ott G Confirmation of the Molecular Classification of Diffuse Large B-Cell Lymphoma by Immunohistochemistry Using a Tissue Microarray. Blood (2004) 103(1):275–82. 10.1182/blood-2003-05-1545 14504078

[29] Rajakariar R Bhattacharyya M Norton A Sheaff M Cavenagh J Raftery MJ Post Transplant T-Cell Lymphoma: A Case Series of Four Patients from a Single Unit and Review of the Literature. Am J Transpl (2004) 4:1534–8. 10.1111/j.1600-6143.2004.00521.x 15307843

[30] Opelz G Döhler B. Lymphomas after Solid Organ Transplantation: A Collaborative Transplant Study Report. Am J Transpl (2004) 4(2):222–30. 10.1046/j.1600-6143.2003.00325.x 14974943

[31] Sampaio MS Cho YW Shah T Bunnapradist S Hutchinson IV. Impact of EpsteinBarr Virus Donor and Recipient Serostatus on the Incidence of post-transplant Lymphoproliferative Disorder in Kidney Transplant Recipients. Nephrol Dial Transpl (2012) 27:2971–9. 10.1093/ndt/gfr769 22273720

[32] Fernberg P Edgren G Adami J Ingvar A Bellocco R Tufveson G Time Trends in Risk and Risk Determinants of Non-hodgkin Lymphoma in Solid Organ Transplant Recipients. Am J Transpl (2011) 11:2472–82. 10.1111/j.1600-6143.2011.03704.x 21883909

[33] Van Leeuwen MT Grulich AE Webster AC McCredie MRE Stewart JH McDonald SP Immunosuppression and Other Risk Factors for Early and Late Non-hodgkin Lymphoma after Kidney Transplantation. Blood (2009) 114:630–7. 10.1182/blood-2009-02-202507 19443660

[34] Na R Laaksonen MA Grulich AE Meagher NS McCaughan GW Keogh AM Iatrogenic Immunosuppression and Risk of Non-hodgkin Lymphoma in Solid Organ Transplantation: A Population-Based Cohort Study in Australia. Br J Haematol (2016) 174:550–62. 10.1111/bjh.14083 27136044

[35] Sampaio MS Cho YW Shah T Bunnapradist S Hutchinson IV. Association of Immunosuppressive Maintenance Regimens with Posttransplant Lymphoproliferative Disorder in Kidney Transplant Recipients. Transplantation (2012) 93:73–81. 10.1097/TP.0b013e31823ae7db 22129761

[36] Romero S Montoro J Guinot M Almenar L Andreu R Balaguer A Post-Transplant Lymphoproliferative Disorders after Solid Organ and Hematopoietic Stem Cell Transplantation. Leuk Lymphoma () 60(1):142–50. 10.1080/10428194.2018.1474462 29966464

[37] Ghobrial IM Habermann TM Maurer MJ Geyer SM Ristow KM Larson TS Prognostic Analysis for Survival in Adult Solid Organ Transplant Recipients with post-transplantation Lymphoproliferative Disorders. J Clin Oncol (2005) 23(30):7574–82. 10.1200/JCO.2005.01.0934 16186599

[38] Tsai DE Bagley S Reshef R Shaked A Bloom RD Ahya V The Changing Face of Adult Posttransplant Lymphoproliferative Disorder: Changes in Histology between 1999 and 2013. Am J Hematol (2018) 93(7):874–81. 10.1002/ajh.25116 29659047

[39] Evens AM Choquet S Kroll-Desrosiers AR Jagadeesh D Smith SM Morschhauser F Primary CNS Posttransplant Lymphoproliferative Disease (PTLD): An International Report of 84 Cases in the Modern Era. Am J Transpl (2013) 13:1512–22. 10.1111/ajt.12211 23721553

[40] Mahale P Shiels MS Lynch CF Engels EA. Incidence and Outcomes of Primary central Nervous System Lymphoma in Solid Organ Transplant Recipients. Am J Transpl (2018) 18:453–61. 10.1111/ajt.14465 PMC5790603 28805292

[41] Cavaliere R Petroni G Lopes MB Schiff D O’Neill BP Plotkin SR Primary central Nervous System post-transplantation Lymphoproliferative Disorder: An International Primary central Nervous System Lymphoma Collaborative Group Report. Cancer (2010) 116:863–70. 10.1002/cncr.24834 20052713 PMC4113953

[42] Montanari F Radeski D Seshan V Alobeid B Bhagat G O’Connor OA. Recursive Partitioning Analysis of Prognostic Factors in post-transplant Lymphoproliferative Disorders (PTLD): A 120 Case Single Institution Series. Br J Haematol (2015) 171(4):491–500. 10.1111/bjh.13621 26250758

[43] Ferreiro JF Morscio J Dierickx D Marcelis L Verhoef G Vandenberghe P Post-Transplant Molecularly Defined Burkitt Lymphomas Are Frequently MYC-Negative and Characterized by the 11q-Gain/loss Pattern. Haematologica (2015) 100:e275–9. 10.3324/haematol.2015.124305 25795716 PMC4486241

[44] Swerdlow SH. T-Cell and NK-Cell Posttransplantation Lymphoproliferative Disorders. Am J Clin Pathol (2007) 127:887–95. 10.1309/LYXN3RGF7D7KPYG0 17509986

[45] Tiede C Maecker-Kolhoff B Klein C Kreipe H Hussein K. Risk Factors and Prognosis in T-Cell Posttransplantation Lymphoproliferative Diseases. Transplantation (2013) 95:479–88. 10.1097/tp.0b013e3182762e07 23296147

[46] Hanson MN Morrison VA Peterson BA Stieglbauer KT Kubic VL McCormick SR Posttransplant T-Cell Lymphoproliferative Disorders - an Aggressive, Late Complication of Solid-Organ Transplantation. Blood (1996) 88:3626–33. 10.1182/blood.v88.9.3626 8896433

[47] Herreman A Dierickx D Morscio J Camps J Bittoun E Verhoef G Clinicopathological Characteristics of Posttransplant Lymphoproliferative Disorders of T-Cell Origin: Single-center Series of Nine Cases and Meta-Analysis of 147 Reported Cases. Leuk Lymphoma (2013) 54:2190–9. 10.3109/10428194.2013.775436 23402267

[48] Koff JL Li JX Zhang X Switchenko JM Flowers CR Waller EK. Impact of the Posttransplant Lymphoproliferative Disorder Subtype on Survival. Cancer (2018) 124(11):2327–36. 10.1002/cncr.31339 29579330 PMC5992039

[49] Barba T Bachy E Maarek A Fossard G Genestier L Anglicheau D Characteristics of T and NK-Cell Lymphomas after Renal Transplantation: a French National Multicentric Cohort Study. Transplantation (2021) 105:1858–68. 10.1097/TP.0000000000003568 33560724

[50] Luskin MR Heil DS Tan KS Choi S Stadtmauer EA Schuster SJ The Impact of EBV Status on Characteristics and Outcomes of Posttransplantation Lymphoproliferative Disorder. Am J Transpl (2015) 15:2665–73. 10.1111/ajt.13324 PMC5726526 25988622

[51] Cho YU Chi HS Jang S Park SH Park CJ. Pattern Analysis of Epstein-Barr Virus Viremia and its Significance in the Evaluation of Organ Transplant Patients Suspected of Having Posttransplant Lymphoproliferative Disorders. Am J Clin Pathol (2014) 141:268–74. 10.1309/AJCP9WYEXKOL9YUV 24436276

[52] Stevens SJC Verschuuren EAM Pronk I Van Der Bij W Harmsen MC The TH Frequent Monitoring of Epstein-Barr Virus DNA Load in Unfractionated Whole Blood Is Essential for Early Detection of Posttransplant Lymphoproliferative Disease in High-Risk Patients. Blood (2001) 97:1165–71. 10.1182/blood.v97.5.1165 11222357

[53] Tsai DE Douglas L Andreadis C Vogl DT Arnoldi S Kotloff R EBV PCR in the Diagnosis and Monitoring of Posttransplant Lymphoproliferative Disorder: Results of a Two-Arm Prospective Trial. Am J Transpl (2008) 8:1016–24. 10.1111/j.1600-6143.2008.02183.x 18312608

[54] Wagner HJ Wessel M Jabs W Smets F Fischer L Offner G Patients at Risk for Development of Posttransplant Lymphoproliferative Disorder: Plasma versus Peripheral Blood Mononuclear Cells as Material for Quantification of Epstein-Barr Viral Load by Using Real-Time Quantitative Polymerase Chain Reaction. Transplantation (2001) 72(6):1012–9. 10.1097/00007890-200109270-00006 11579293

[55] Ferla V Rossi FG Goldaniga MC Baldini L. Biological Difference between Epstein–Barr Virus Positive and Negative Post-transplant Lymphoproliferative Disorders and Their Clinical Impact. Front Oncol (2020) 10:506. 10.3389/fonc.2020.00506 32457824 PMC7225286

[56] Kinch A Baecklund E Backlin C Ekman T Molin D Tufveson G A Population-Based Study of 135 Lymphomas after Solid Organ Transplantation: The Role of Epstein-Barr Virus, Hepatitis C and Diffuse Large B-Cell Lymphoma Subtype in Clinical Presentation and Survival. Acta Oncol (2014) 53(5):669–79. 10.3109/0284186X.2013.844853 24164103

[57] Santarsieri A Rudge JF Amin I Gelson W Parmar J Pettit S Incidence and Outcomes of post-transplant Lymphoproliferative Disease after 5365 Solid-Organ Transplants over a 20-year Period at Two UK Transplant Centres. Br J Haematol (2022) 197:310–9. 35235680 10.1111/bjh.18065

[58] Jagadeesh D Tsai DE Wei W Bustamante JA Wagner-Johnston ND Berg S Post-Transplant Lymphoproliferative Disorder (PTLD) after Solid Organ Transplant (SOT): A Multicenter Real World Analysis (RWA) of 877 Patients (Pts) Treated in the Modern Era. J Clin Oncol (2020) 38(15):e20026. 10.1200/jco.2020.38.15_suppl.e20026

[59] Reshef R Vardhanabhuti S Luskin MR Heitjan DF Hadjiliadis D Goral S Reduction of Immunosuppression as Initial Therapy for Posttransplantation Lymphoproliferative Disorder. Am J Transpl (2011) 11:336–47. 10.1111/j.1600-6143.2010.03387.x PMC3079420 21219573

[60] Leyssens A Dierickx D Verbeken EK Tousseyn T Verleden SE Vanaudenaerde BM Post-Transplant Lymphoproliferative Disease in Lung Transplantation: A Nested Case-Control Study. Clin Transpl (2017) 31(7):e12983. 10.1111/ctr.12983 28383790

[61] Burns DM Clesham K Hodgson YA Fredrick L Haughton J Lannon M Real-world Outcomes with Rituximab-Based Therapy for Posttransplant Lymphoproliferative Disease Arising after Solid Organ Transplant. Transplantation (2020) 104(12):2582–90. 10.1097/TP.0000000000003183 33104308

