# Supplementary material for: Characteristics and Outcome of Post-Transplant Lymphoproliferative Disorders After Solid Organ Transplantation: A Single Center Experience of 196 Patients Over 30 Years
Source: Transpl Int. 2022 Dec 14;35:10707. doi: 10.3389/ti.2022.10707 (PMC9794588; doi:10.3389/ti.2022.10707)
Supplement: Supplementary file 1 [file DataSheet1.PDF]

Supplementary materials

Statistical methodology

Kaplan – Meier estimates were used to create survival curves for overall survival (OS) and relapse-free survival (RFS), the latter in the group of patients with complete response (CR). Nelson-Aalen estimates were used to construct a curve for the percentage PLTD deaths, considering deaths due to other causes as a competing risk.

The relation of EBV and year of PTLD diagnosis with OS, PTLD-related death, complete response, relapse and RFS was evaluated. Univariable as well as multivariable Cox or logistic regression models were used, depending on the type of outcome. Given that PTLD-unrelated death is a competing risk for PTLD-related death, results from Fine and Gray models were verified as well. The following variables were a priori defined to be considered as confounders in the multivariable models when evaluating the independent effect of EBV and year of diagnosis: IPI score, sex, transplanted organ (kidney/liver/heart/lung), graft organ involvement (yes/no), monomorphic (yes/no), central nervous system (CNS) involvement (no or no suspicion of CNS involvement/CNS involved, primary/CNS involved, not primary), CD20 status (positive/negative) and serum albumin level (within or below normal limits). Although age at PTLD diagnosis is a component in the IPI score it was added because of its earlier reported strong impact on OS. The other factors included in the IPI (ECOG PS, extranodal sites, stage, LDH), were not repeated in the multivariable analysis. The same list of potential confounders was used for each of the outcomes.

For some of the considered predictors there was a proportion of patients with a missing value resulting in 12.4% of patients with at least one variable missing. To handle the presence of the missing values, a multiple imputation approach was used. Multivariate imputation was performed using the fully conditional specification approach.[1] In this approach, for each of the variables with a missing value, a regression model is specified using all other predictors and outcome variables as covariates. Depending on the variable with missing information, a linear regression or binary logistic regression model was

used. The process was reiterated (one iteration consists of one cycle through all variables) until convergence to the multivariate distribution was obtained. Twenty complete datasets were created and the multivariable regression models (Logistic, Cox or Fine and Gray) was fitted in each of the datasets. The results of the 20 analyses performed on the 20 completed datasets were combined using Rubin's rule.[2] The predictors considered in this model were determined based on a backward stepwise selection procedure with 0.157 as critical level for the p-value. This critical value corresponds to the use of the Aikake Information Criterion for model selection. With Aikake Information Criterion we require that the increase in model χ^2 has to be larger than two times the degrees of freedom. This strategy provides a trade-off between erroneous inclusion and exclusion of covariates in a prediction model.[3] 'Stepwise' refers to the fact that after each backward step, all variables which were removed in earlier steps are checked again one by and one and those with $p<0.157$ were entered again in the model. The model reduction was performed on a stacked dataset consisting of the multiply imputed data, using a weighting scheme to account for the fraction of missing data for each covariate.[4] Given the small number of relapses, no multivariable model was constructed for this outcome.

Restricted cubic splines were used to allow non-linearity in the relation with year of PTLD diagnosis. However, for all the outcomes except RFS (univariable setting) the assumption of linearity was plausible and therefore linearity was assumed in the reported models.

If the two predictors of interest (EBV, year of PTLD diagnosis) were not retained in the multivariable model, a sensitivity analysis was performed adding them again to the final model.

P-values smaller than 0.05 were considered significant. No corrections for multiple testing were considered. All analyses have been performed using SAS software, version 9.4 of the SAS System for Windows. Copyright © 2002 SAS Institute Inc. SAS and all other SAS Institute Inc. product or service names are registered trademarks or trademarks of SAS Institute Inc., Cary, NC, USA.

References

1. Van Buuren S. Multiple imputation of discrete and continuous data by fully conditional

specification. *Stat Methods Med Res*. 2007;16(3):219-242. doi:10.1177/0962280206074463

2. Rubin DB, Wiley J, York N, Brisbane C, Singapore T. *Multiple Imputation for Nonresponse in Surveys*.; 1987.

3. Sauerbrei W. The Use of Resampling Methods to Simplify Regression Models in Medical Statistics. *J R Stat Soc Ser C (Applied Stat*. 1999;48(3):313-329. doi:10.1111/1467-9876.00155

4. Wood AM, White IR, Royston P. How should variable selection be performed with multiply imputed data? *Stat Med*. 2008;27(17):3227-3246. doi:10.1002/sim.3177
